# A Case of Persistent KSHV Viremia in the Context of HIV, SARS-CoV-2, and Other Co-Infections

**DOI:** 10.3390/tropicalmed10020053

**Published:** 2025-02-10

**Authors:** Humaira Lambarey, Melissa J. Blumenthal, Prishanta Chinna, Vincent N. Naude, Lauren Jennings, Catherine Orrell, Georgia Schäfer

**Affiliations:** 1 International Centre for Genetic Engineering and Biotechnology (ICGEB), Cape Town 7925, South Africa; lmbhum001@myuct.ac.za (H.L.); melissa.blumenthal@icgeb.org (M.J.B.); pllpri021@myuct.ac.za (P.C.); vnnaude@gmail.com (V.N.N.); 2 Institute of Infectious Disease and Molecular Medicine (IDM), Faculty of Health Sciences, University of Cape Town, Cape Town 7925, South Africa; catherine.orrell@hiv-research.org.za; 3 Division of Medical Biochemistry and Structural Biology, Department of Integrative Biomedical Sciences, Faculty of Health Sciences, University of Cape Town, Cape Town 7925, South Africa; 4 Desmond Tutu HIV Centre and Department of Medicine, Faculty of Health Sciences, University of Cape Town, Cape Town 7925, South Africa; lauren.jennings@hiv-research.org.za; 5 Wellcome Centre for Infectious Diseases Research in Africa, University of Cape Town, Cape Town 7925, South Africa

**Keywords:** Kaposi’s sarcoma-associated herpesvirus (KSHV or HHV-8), human immunodeficiency virus (HIV), people living with HIV (PLWH), severe acute respiratory syndrome coronavirus 2 (SARS-CoV-2), COVID-19

## Abstract

Despite the high prevalence of latent Kaposi’s sarcoma-associated herpesvirus (KSHV) infections in patients from endemic areas with a high human immunodeficiency virus (HIV) prevalence, KSHV lytic reactivation in the context of other co-infections is not well understood. Lytic KSHV infections can contribute to severe inflammatory symptoms and KSHV-associated pathogenesis. We have previously reported on KSHV reactivation upon severe acute respiratory syndrome coronavirus 2 (SARS-CoV-2) exposure in a non-hospitalised cohort of people living with HIV (PLWH). From this cohort, we identified a 34-year-old male who presented for routine HIV care in May 2021 with an unusually high KSHV viral load (VL) of 189,946.3 copies/10^6^ cells, before SARS-CoV-2 infection. The patient was invited into a 2-year follow-up study where his peripheral blood was analysed for selected virological, clinical, and inflammatory parameters every 6 months. He remained highly viremic for KSHV throughout the 2-year study period, during which he was infected with SARS-CoV-2 and developed disseminated tuberculosis, with steadily increasing levels of the inflammatory markers C-reactive protein (CRP), and interleukin-6 (IL-6). His HIV VL remained controlled (<1000 copies/mL) and his CD4 count bordered immunosuppression (±200 cells/µL), suggesting some responsiveness to antiretroviral treatment (ART). However, the patient’s uncontrolled lytic KSHV infection may increase his risk for developing a KSHV-associated pathology manifesting with inflammation which should be closely monitored beyond the study period.

## 1. Introduction

Kaposi’s sarcoma-associated herpesvirus (KSHV, or HHV-8) is an oncogenic gammaherpesvirus highly prevalent in Sub-Saharan Africa and the causative agent of Kaposi’s sarcoma (KS), multicentric Castleman disease (MCD), primary effusion lymphoma (PEL), and the recently described KSHV-associated inflammatory cytokine syndrome (KICS) [1,2]. These pathologies primarily occur in the context of co-infection with human immunodeficiency virus (HIV), which also displays a high seroprevalence in Sub-Saharan Africa.

KSHV infections mainly occur early in life through salivary transmission among family members, especially in children residing in endemic areas [3]. Infected individuals experience a life-long latent infection which can be interrupted by intermittent lytic reactivation triggered by various stimuli such as other infections, inflammation, or oxidative stress [4]. While both latent and lytic lifecycle phases contribute to KSHV-associated pathologies, the introduction of antiretroviral therapy (ART) has led to a substantial reduction in KS incidence rates in South Africa and several other regions in Sub-Saharan Africa [5,6]. ART administration also supports treatment outcomes of PEL [1], while the incidence of KSHV-associated MCD has increased from the pre-ART to the current ART era [7].

Although rare compared to KS, inflammatory manifestations associated with KSHV infection such as MCD and KICS in people living with HIV (PLWH) with well-preserved immune function have gained increasing attention [8,9,10]. These inflammatory conditions are linked to lytic KSHV reactivation, which is not only characterised by elevated KSHV viral load (VL) in the blood, but also by the expression of lytic KSHV-associated genes which encode for cytokines or induce host cytokine production [9]. Moreover, patients with MCD or KICS have been reported to present with similar laboratory and clinical abnormalities, with limited treatment options and high mortality [8,9]. It is, therefore, important to better understand the underlying triggers of KSHV lytic reactivation and to monitor patients with high KSHV VL in order to assess risks for development of KSHV-associated pathologies and to evaluate and improve treatment strategies, particularly in HIV healthcare facilities.

With the aim to assess the impact of the COVID-19 pandemic on KSHV infection dynamics in non-hospitalised adult PLWH attending ART services in South Africa, we have previously reported a cross-sectional study (n = 407) conducted at a community health centre antiretroviral clinic (Desmond Tutu HIV Centre) in Gugulethu, a densely populated, low-income, peri-urban area outside Cape Town, South Africa, from October 2020 to April 2023 [11]. The majority of patients (85.0%) were receiving ART at the time of recruitment, had a CD4 count of <350 cells/µL, and a median HIV VL of 49 copies/mL (IQR 1–1426 copies/mL). Exposure to severe acute respiratory syndrome coronavirus 2 (SARS-CoV-2) infection (as assessed by SARS-CoV-2 serology) was as high as 76.2% at the start of recruitment (before the roll-out of COVID-19 vaccinations), and increased to 94.9% at the end of the recruitment period, encompassing self-reported COVID-19 vaccination and exposure to multiple COVID-19 infection waves [11]. The overall KSHV seroprevalence in the cohort was 53.5%, and we identified a significant association between KSHV VL (as a proxy for KSHV reactivation) and SARS-CoV-2 exposure in COVID-19 unvaccinated patients, suggesting that multiple and repeated exposure to SARS-CoV-2 in unvaccinated patients may have lasting effects on KSHV reactivation, particularly in the context of PLWH [11].

**Figure 1:**
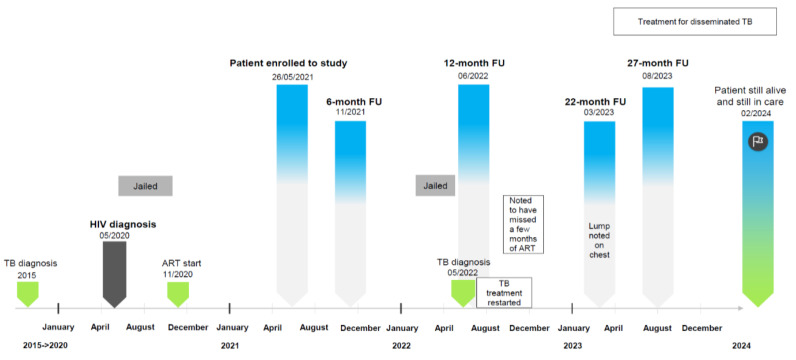
The patient’s medical history and course of events from 2015 to 2024. Please refer to the text for details.

From this cohort, we identified one patient who had an unusually high KSHV VL at recruitment compared to the remainder of the cohort. This patient, a 34-year-old HIV-positive black male, presented for general check-up on 26 May 2021 and was enrolled into this observational study on the same day. The patient was not vaccinated against COVID-19 and self-reportedly never received COVID-19 vaccination throughout the 2-year follow-up (FU) study period (Figure 1 and Table 1).

## 2. Materials and Methods

Recruitment of this patient was conducted in the context of a larger study which has been published recently [11]. Ethics approval for the parental study was obtained from the University of Cape Town’s Health Sciences Research Ethics Committee (HREC 134/2020). Written informed consent was obtained from all the participants involved in the parental study, including the case reported here.

Clinical and demographic information and peripheral blood samples were collected at the time of enrolment as well as each FU visit, together with information on COVID-19 vaccination status and self-reported symptoms. Laboratory parameters such as absolute CD4 count, HIV VL, C-reactive protein (CRP), full blood count, and differential cell count were analysed by the National Health Laboratory Services (NHLS), with ART information obtained from pharmacy records. In-house assays were performed for IL-6 concentration in the peripheral blood as well as SARS-CoV-2 serology, KSHV serology, and KSHV VL, as described previously [11]. EBV serology was determined using the Anti-Epstein Barr virus (EBV-EBNA) IgG Human ELISA kit according to the manufacturers’ protocol (Abcam, Cambridge, England, UK), while EBV VL was determined as described in [12].

A Pearson correlation test was employed to assess linear relationships between KSHV VL and other linear variables, and the resultant correlation coefficients are presented in a correlation matrix.

## 3. Results

### 3.1. General Description of the Case in the Context of Routine HIV Clinical Care

At presentation, the patient reported no symptoms associated with either KSHV or SARS-CoV-2 infection or any other signs of pathologies or abnormalities. His past medical history revealed a previous tuberculosis (TB) diagnosis in 2015 and a positive HIV diagnosis in May 2020 (with a World Health Organization (WHO) stage 2 clinical stage of HIV disease). The patient did not start ART immediately upon his HIV diagnosis due to imprisonment, but only in November 2020 where his regimen consisted of tenofovir/emtricitabine/efavirenz (TDF/FTC/EFV). However, his ART regimen was changed to tenofovir/lamivudine/dolutegravir/ (TDF/3TC/DTG) in July 2021 due to changes in national ART guidelines. In June 2022, the patient’s ART regimen was changed to abacavir/lamivudine/dolutegravir (ABC/3TC/DTG) because of abnormal renal function (indicated by high creatinine levels, Table 1). He has since remained on this regimen.

Following study enrolment in May 2021, the patient was invited for FU visits at the 6-, 12-, 18-, and 24-month marks, at which times clinical examinations were conducted and peripheral blood analysed for study-relevant variables (Figure 1 and Table 1). It should be noted that the patient missed his 18- and 24-month FU visits and presented at the 22- and 27-month marks instead (Figure 1 and Table 1).

**Figure 2:**
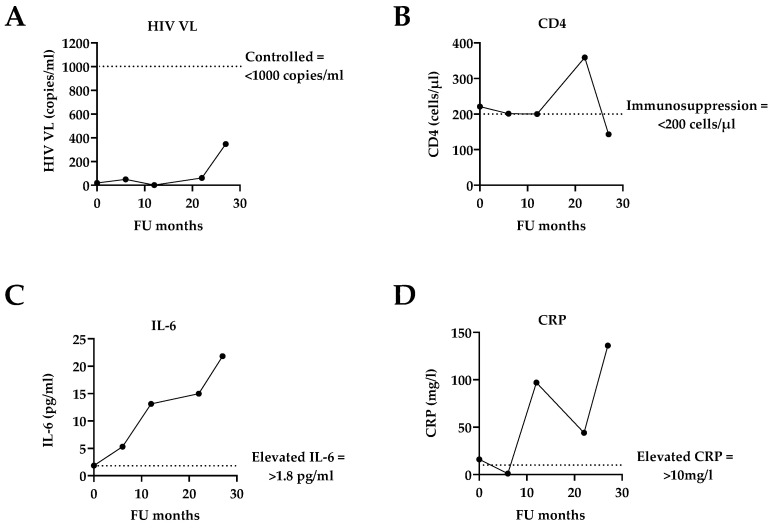
Selected HIV and inflammatory variables throughout the 2-year FU period. (**A**) HIV VL; (**B**) CD4 count; (**C**) IL-6 concentration; (**D**) CRP concentration. The dotted lines indicate the clinical threshold for the individual variables.

At the time of the patient’s enrolment into the study, he had an HIV VL of <20 copies/mL which remained well below the clinical threshold of 1000 copies/mL, indicating a controlled HIV VL [13] throughout the FU period. His CD4 count at enrolment was 221 cells/µL, bordering the criteria for immunosuppression (CD4 ≤ 200 cells/µL, [13]), but remained relatively constant throughout (Table 1 and Figure 2). However, we noted some severe fluctuations in the patient’s weight and increasing self-reported symptoms at presentation over the course of the 2-year study which may have coincided with his imprisonment in February 2022 and his clinical TB diagnosis in May 2022. He was also noted to be nonadherent to his TB and ART medication and was diagnosed with a lump on his chest by abdominal ultrasound in March 2023 which initiated further treatment for disseminated TB. Some abnormal blood results over the study period included increased levels of creatinine (indicating renal impairment as mentioned above) as well as steadily increasing concentrations of the inflammatory markers interleukin-6 (IL-6) and C-reactive protein (CRP), reaching 21.83 mg/L and 136 mg/L, respectively (Table 1 and Figure 2), indicating severe inflammation.

As of February 2024, the patient was still in care and presenting at the clinic for regular check-ups.

### 3.2. Assessment of Co-Infections: SARS-CoV-2, KSHV, and EBV

**Figure 3:**
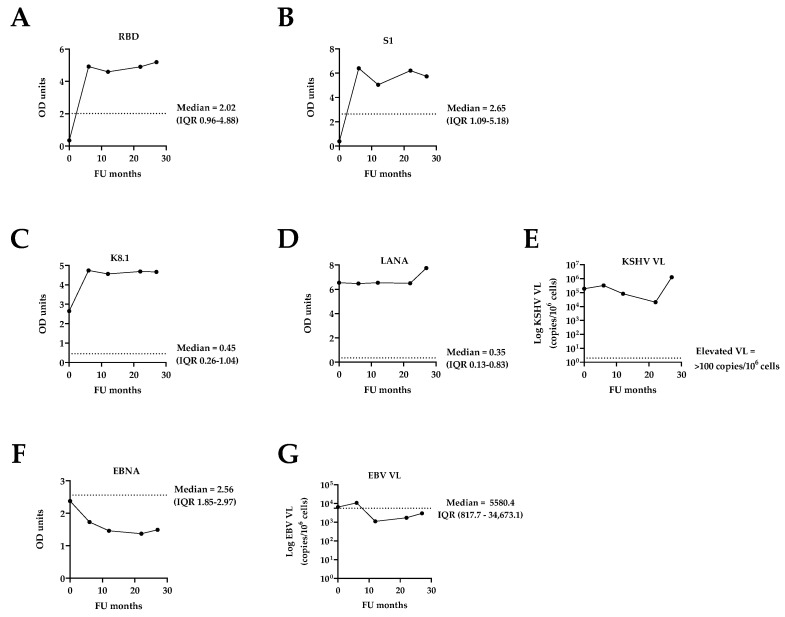
Selected virological variables throughout the 2-year FU period. (**A**) SARS-CoV-2 RBD and (**B**) SARS-CoV-2 S1 IgG antibody levels; (**C**) KSHV K8.1 and (**D**) KSHV LANA antibody levels; (**E**) KSHV VL; (**F**) EBV EBNA serology; (**G**) EBV VL. The dotted lines indicate the median with the IQR of the entire cohort (n = 407) [11], except for (**E**) where the dotted line indicates the threshold for an elevated KSHV VL = 100 copies/10^6^ cells [8,9].

Over the course of the study, the patient increasingly reported symptoms which may have been associated with COVID-19 (Table 1); however, these symptoms could have overlapped with other chronic diseases such as HIV/AIDS, TB, and/or potentially be attributable to an active KSHV infection. Initially, SARS-CoV-2 serology was negative (RBD = 0.35 and S1 = 0.39 OD units). By the 6-month FU, SARS-CoV-2 antibodies had considerably increased (RBD = 4.92 and S1 = 6.40 OD units) and stayed consistently high and well above the median ODs of the entire cohort throughout the FU period (Table 1 and Figure 3A,B). As self-reportedly not having received the COVID-19 vaccine throughout the FU period, these high levels of antibodies are indicative of natural SARS-CoV-2 infection(s).

Compared to the whole cohort [11], the patient displayed considerably high KSHV serology at the initial enrolment with lytic antigen (K8.1) and latent antigen (LANA) levels of 2.65 and 6.54 OD units, respectively. While K8.1 had increased to 4.74 OD units by the 6-month FU and remained relatively constant afterwards, LANA titers remained at a similar level throughout the entire FU period. Both antibody titers were well above the median antibody levels of the entire cohort (Table 1, Figure 3C,D). These serological observations were further supported by the patient’s KSHV virology: he displayed an unusually high KSHV VL of 189,946.3 copies/10^6^ cells at enrolment, which almost doubled to 325,019.4 copies/10^6^ cells by the 6-month FU, followed by some fluctuations, i.e., 83,107.2, 20,434.6, and 1,278,350.5 copies/10^6^ cells, among the 12-, 22-, and 27-month FUs, respectively (Table 1 and Figure 3E). These values were clearly above the threshold for elevated KSHV VL of 100 copies/10^6^ cells, as previously defined [8,9], and substantially higher than the median KSHV VL of the entire cohort (Figure 3E).

In addition, the infection dynamics of the related oncogenic gammaherpesvirus Epstein–Barr virus (EBV) were assessed. In contrast to KSHV, EBV is ubiquitous worldwide and has been linked to infectious mononucleosis, Burkitt’s lymphoma, Hodgkin’s lymphoma, and other lymphoproliferative diseases which particularly occur in the context of HIV co-infection [14,15,16]. Both KSHV and EBV can be lytically reactivated by various stimuli such as immunosuppression, immunodeficiency, inflammation, and the presence of different antigens due to new infections and co-infections [17,18,19]. However, both EBV serology, as determined by antibodies against the EBV nuclear antigen (EBNA), and EBV VL remained at or below the respective median values of the entire cohort throughout the study period (Table 1 and Figure 3F,G).

## 4. Discussion

Reactivation of gammaherpesviruses, such as KSHV and EBV, from latency has been suggested to be triggered by various stimuli, which may have consequences for the development of associated pathologies [4]. However, the impact of the COVID-19 pandemic on gammaherpesvirus reactivation, particularly in endemic areas and in the context of HIV co-infection, is not clear. While some studies on hospitalised critically ill COVID-19 patients have reported on herpesvirus reactivation [20,21,22,23,24,25,26,27,28], we have recently shown that high and repeated exposure to SARS-CoV-2 also increases the risk of KSHV reactivation in non-hospitalised PLWH who are COVID-19 unvaccinated [11]. While the majority of patients with reactivated KSHV displayed a very low VL (which we defined as “detectable but not quantifiable” [11]), only a few patients stood out with an elevated KSHV VL above the threshold of 100 copies/10^6^ cells [8,9]. One such patient was enrolled into the parent study with a KSHV VL of 189,946.3 copies/10^6^ cells and was monitored over the course of 2 years with regard to gammaherpesvirus infection dynamics in the light of potential risks for the development of KSHV-associated pathologies.

While the patient was SARS-CoV-2-seronegative at enrolment, he was most likely infected during the first 6 months of this study, as his SARS-CoV-2 antibody titers increased substantially in the absence of COVID-19 vaccination. It is, therefore, unlikely that SARS-CoV-2 exposure caused KSHV reactivation in this case, as the patient presented with a highly elevated KSHV VL before SARS-CoV-2 infection. Also, his EBV VL was not affected following SARS-CoV-2 exposure and remained quantitatively within the range of the entire cohort throughout the study period.

Although the patient was in HIV care displaying a well-controlled HIV VL, his CD4 count bordered immunosuppression throughout the study. This may be explained by the patient’s living circumstances (such as imprisonment) and/or by the patient missing some ART and TB treatments. Additionally, his severe weight fluctuations, indicating malnutrition, his steadily increasing levels of inflammatory markers and increasing self-reported symptoms at presentation (which may be a reflection of his TB disease) may have contributed to an overall state which supported active KSHV replication, detectable as high VL in the blood. Although our observational study design did not identify the cause of the patient’s persistent and uncontrolled KSHV viremia, correlation analysis indicated some positive correlations between KSHV VL and HIV VL over the study period, concomitant with a negative correlation with CD4 count, indicating that the KSHV VL increased with a worsening HIV infection (Supplementary Figure S1). Similarly, there was a positive correlation with inflammatory markers, CRP and IL-6, and creatinine and a negative correlation with platelet count, indicative of a rising inflammation, a worsening kidney function, and thrombocytopenia, potentially attributable to worsening HIV and general condition.

It is important to note that the patient’s ART treatment (while clinically controlling his HIV infection) did not influence his KSHV VL. However, it should also be noted that no specific interventions targeting his KSHV viremia took place during the course of this observational study. Although cases of uncontrolled KSHV viremia are rare in the context of ART, it has been reported that KSHV-associated MCD (which is characterised by elevated KSHV VL, accompanied by inflammation) has not declined in number since the onset of the ART era [7]. Interestingly, a recent case study reporting on KICS in the context of a well-controlled HIV infection demonstrated a good response to antiviral and anti-inflammatory medication, reducing KSHV VL and inflammatory markers [10]. The EBV VL remained low and unaffected by this patient’s KSHV viremia [10]. Although rare (but most likely underdiagnosed due to invasive diagnostical procedures), the diagnosis and treatment of KICS and other pathologies associated with lytic KSHV infection highlights the importance of assessing and monitoring KSHV viremia in HIV care, particularly in the context of unexplained inflammation. This case report highlights the need to adapt the current KICS case definition to the context of Sub-Saharan Africa [29], where TB is highly prevalent and currently excludes a patient from a KICS diagnosis and, therefore, treatment, while co-occurrence may potentially be at play.

## 5. Conclusions and Perspective

We have previously provided strong clinical evidence that elevated KSHV VL is associated with morbidity and mortality in the context of inflammatory conditions such as TB [30] or severe COVID-19 [20], respectively. We have also shown that KS patients with extremely high blood KSHV VL have a worse disease outcome [31], potentially due to concurrent KSHV-associated conditions such as MCD or KICS which require invasive diagnosis and are, therefore, often left undiagnosed in the presence of KS and/or TB. Although the patient in our study was not diagnosed with an inflammatory condition linked to a KSHV-associated disease, he should be monitored beyond the study period for KSHV-related parameters and inflammatory markers as part of his HIV care. This is particularly relevant in low-resource settings with high HIV/KSHV prevalence, where invasive diagnostic procedures are often not feasible. A heightened awareness of KSHV viremia in the context of inflammation is critically needed to inform prognostic implications and clinical management of potential KSHV-associated pathologies manifesting with inflammation.

## Figures and Tables

**Table 1:** Clinical and virological parameters of the patient. Indicated in bold are abnormal values for sodium (<136 mmol/L), creatinine (>104 µmol/L), CD4 count (≤200 cells/µL), elevated KSHV VL (>100 copies/10^6^ cells), elevated EBV VL (>median of parental cohort = 5580.4 copies/10^6^ cells), IL-6 (>1.8 pg/mL), and CRP (>10 mg/L). * Lower than the detectable limit.

FU Month		0	6	12	22	27
Symptoms at presentation (self-reported)		None	Respiratory symptoms, coughing, weight loss, night sweats, arthralgia, myalgia	Fatigue, oedema, cachexia, respiratory symptoms, coughing, gastrointestinal disturbance, altered mental state, weight loss, night sweats, neuropathy, radiographic abnormalities, arthralgia, myalgia	Oedema, cachexia, gastrointestinal disturbance, weight loss, night sweats, arthralgia, myalgia, neuropathy with pain (post TB treatment)	Respiratory symptoms, coughing, night sweats, arthralgia, myalgia
Weight (kg)		No information	61.6	43	54	45
WHO stage		2	2	3	4	4
ART regimen		TDF/FTC/EFV	TDF/FTC/EFV	TDF/3TC/DTG	ABC/3TC/DTG	ABC/3TC/DTG
TB diagnosis		No	No	Yes	Yes	Yes
COVID-19 vaccination (self-reported)		No	No	No	No	No
**Laboratory blood analysis (chemical pathology and haematology):**						
Sodium (mmol/L)		**135**	136	140	138	**135**
Creatinine (µmol/L)		74	76	**115**	**175**	**150**
Platelet count (×10^9^/L)		269	184	275	245	162
Absolute CD4 (cells/µL)		221	201	**200**	359	**143**
**Virology (serology and VL):**						
HIV VL (copies/mL)		<20	<50	1 *	61	347
SARS-CoV-2 serology (OD)	RBD	0.35	4.92	4.59	4.90	5.19
	S1	0.39	6.40	5.05	6.21	5.74
KSHV serology (OD)	K8.1	2.65	4.74	4.56	4.69	4.66
	LANA	6.54	6.48	6.54	6.50	7.75
EBV serology–EBNA (OD units)		2.38	1.73	1.46	1.37	1.49
KSHV VL (copies/10^6^ cells)		**189,946.3**	**325,019.4**	**83,107.2**	**20,434.6**	**1,278,350.5**
EBV VL (copies/10^6^ cells)		**6450.0**	**10,800.0**	1120.0	1730.0	3000.0
**Inflammatory markers:**						
IL-6 (pg/mL)		**1.96**	**5.28**	**13.11**	**14.99**	**21.83**
CRP (mg/L)		**16**	1	**97**	**44**	**136**

## Data Availability

The data that support the findings of this article are openly available at PubMed (https://pubmed.ncbi.nlm.nih.gov/).

## Supplementary Materials

The following supporting information can be downloaded at: https://www.mdpi.com/article/10.3390/tropicalmed10020053/s1, Figure S1: Correlation matrix of clinical variables recorded over the 2-years study period. Correlation coefficients were generated by Pearson correlation testing. Statistically significant correlations (*p* < 0.05) are highlighted in bold.
