# Supplementary material for: A Case of Persistent KSHV Viremia in the Context of HIV, SARS-CoV-2, and Other Co-Infections
Source: Trop Med Infect Dis. 2025 Feb 10;10(2):53. doi: 10.3390/tropicalmed10020053 (PMC11860674; doi:10.3390/tropicalmed10020053)
Supplement: Supplementary file 1 [file tropicalmed-10-00053-s001.zip › tropicalmed-3466435-supplementary.pdf]

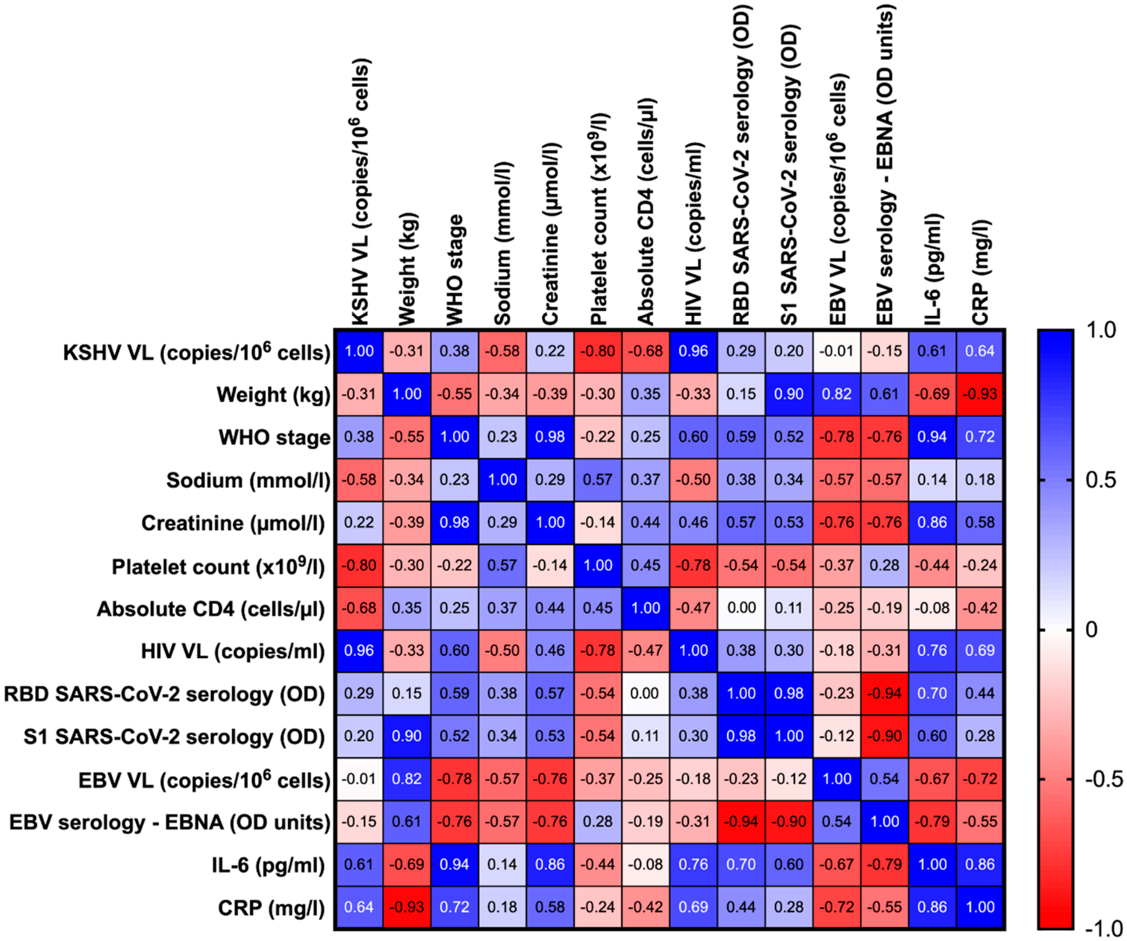

Supplementary Figure S1. Correlation matrix of clinical variables recorded over the 2-years study period. Correlation coefficients were generated by Pearson correlation testing. Statistically significant correlations ($p < 0.05$) are highlighted in bold.
